# Supplementary material for: Modeling glioblastoma heterogeneity as a dynamic network of cell states
Source: Mol Syst Biol. 2021 Sep 16;17(9):e10105. doi: 10.15252/msb.202010105 (PMC8444284; doi:10.15252/msb.202010105)
Supplement: Supplementary file 6 — Source Data for Figure 5 [file MSB-17-e10105-s004.zip › Figure5A_sourcedata/GSEA_3071/nosigdb_stateIII.GseaPreranked.1622795155562/DOROSZKO_U3054MG_CLUSTER_0.html]

Details for gene set DOROSZKO\_U3054MG\_CLUSTER\_0[GSEA]

|  || Dataset | state33071 |
| Phenotype | NoPhenotypeAvailable |
| Upregulated in class | na\_neg |
| GeneSet | DOROSZKO\_U3054MG\_CLUSTER\_0 |
| Enrichment Score (ES) | -0.5534375 |
| Normalized Enrichment Score (NES) | -2.8848677 |
| Nominal p-value | 0.0 |
| FDR q-value | 0.0 |
| FWER p-Value | 0.0 |
Table: GSEA Results Summary

  

Fig 1: Enrichment plot: DOROSZKO\_U3054MG\_CLUSTER\_0      
 Profile of the Running ES Score & Positions of GeneSet Members on the Rank Ordered List

  

| PROBE | GENE SYMBOL | GENE\_TITLE | RANK IN GENE LIST | RANK METRIC SCORE | RUNNING ES | CORE ENRICHMENT || 1 | MBP |  |  | 493 | -0.254 | -0.1158 | No |
| 2 | TMEM132B |  |  | 578 | -0.258 | -0.1260 | No |
| 3 | GPC4 |  |  | 593 | -0.259 | -0.1183 | No |
| 4 | CPM |  |  | 637 | -0.261 | -0.1179 | No |
| 5 | BCHE |  |  | 867 | -0.272 | -0.1649 | No |
| 6 | SLC44A1 |  |  | 1119 | -0.286 | -0.2170 | No |
| 7 | COBL |  |  | 1149 | -0.287 | -0.2118 | No |
| 8 | FIBIN |  |  | 1458 | -0.304 | -0.2778 | No |
| 9 | CREB5 |  |  | 1470 | -0.304 | -0.2673 | No |
| 10 | PPP1R9A |  |  | 1472 | -0.304 | -0.2542 | No |
| 11 | YPEL2 |  |  | 1716 | -0.320 | -0.3027 | No |
| 12 | ADAM23 |  |  | 1967 | -0.334 | -0.3524 | No |
| 13 | TTYH1 |  |  | 2397 | -0.365 | -0.4469 | No |
| 14 | SEMA6D |  |  | 2812 | -0.400 | -0.5359 | Yes |
| 15 | SFRP1 |  |  | 2839 | -0.402 | -0.5249 | Yes |
| 16 | ALCAM |  |  | 2853 | -0.403 | -0.5106 | Yes |
| 17 | CHRDL1 |  |  | 2917 | -0.410 | -0.5088 | Yes |
| 18 | PRUNE2 |  |  | 2966 | -0.416 | -0.5030 | Yes |
| 19 | SLC6A8 |  |  | 3004 | -0.420 | -0.4940 | Yes |
| 20 | P2RX7 |  |  | 3016 | -0.422 | -0.4784 | Yes |
| 21 | WWP2 |  |  | 3075 | -0.428 | -0.4745 | Yes |
| 22 | PLCB1 |  |  | 3189 | -0.441 | -0.4842 | Yes |
| 23 | COL15A1 |  |  | 3317 | -0.464 | -0.4966 | Yes |
| 24 | PCSK1N |  |  | 3346 | -0.470 | -0.4832 | Yes |
| 25 | MCAM |  |  | 3353 | -0.472 | -0.4640 | Yes |
| 26 | PCBP4 |  |  | 3381 | -0.476 | -0.4501 | Yes |
| 27 | OLIG1 |  |  | 3486 | -0.503 | -0.4549 | Yes |
| 28 | CKB |  |  | 3572 | -0.536 | -0.4532 | Yes |
| 29 | CTHRC1 |  |  | 3591 | -0.544 | -0.4340 | Yes |
| 30 | TUBB2B |  |  | 3594 | -0.544 | -0.4106 | Yes |
| 31 | SOX2 |  |  | 3613 | -0.555 | -0.3910 | Yes |
| 32 | ERBB3 |  |  | 3617 | -0.558 | -0.3672 | Yes |
| 33 | CD9 |  |  | 3634 | -0.567 | -0.3465 | Yes |
| 34 | SHC3 |  |  | 3642 | -0.573 | -0.3231 | Yes |
| 35 | SOX6 |  |  | 3675 | -0.596 | -0.3052 | Yes |
| 36 | SLAIN1 |  |  | 3718 | -0.631 | -0.2884 | Yes |
| 37 | PMEPA1 |  |  | 3737 | -0.657 | -0.2642 | Yes |
| 38 | FAM181B |  |  | 3760 | -0.686 | -0.2398 | Yes |
| 39 | TNFAIP6 |  |  | 3780 | -0.713 | -0.2134 | Yes |
| 40 | NCAM1 |  |  | 3821 | -0.773 | -0.1898 | Yes |
| 41 | CDH2 |  |  | 3840 | -0.813 | -0.1587 | Yes |
| 42 | SORL1 |  |  | 3851 | -0.842 | -0.1244 | Yes |
| 43 | CADM1 |  |  | 3856 | -0.851 | -0.0881 | Yes |
| 44 | COL9A3 |  |  | 3896 | -1.084 | -0.0506 | Yes |
| 45 | PTPRZ1 |  |  | 3913 | -1.342 | 0.0041 | Yes |
Table: GSEA details [plain text format]

  

Fig 2: DOROSZKO\_U3054MG\_CLUSTER\_0: Random ES distribution      
 Gene set null distribution of ES for **DOROSZKO\_U3054MG\_CLUSTER\_0**
